# Antenatal depression is associated with pregnancy-related anxiety, partner relations, and wealth in women in Northern Tanzania: a cross-sectional study

**DOI:** 10.1186/s12905-015-0225-y

**Published:** 2015-09-02

**Authors:** Mechtilda Rwakarema, Shahirose S. Premji, Elias Charles Nyanza, Ponsiano Riziki, Luz Palacios-Derflingher

**Affiliations:** St. Augustine University of Tanzania, P.O. Box 307, Mwanza, Tanzania; Faculty of Nursing, University of Calgary, 2500 University Drive NW, Calgary, Alberta T2N 1N4 Canada; Faculty of Medicine, Department of Community Health Sciences, University of Calgary, 2500 University Drive NW, Calgary, Alberta T2N 1N4 Canada; School of Public Health, Catholic University of Health and Allied Sciences, P.O. Box 1464, Bugando Area, Mwanza, Tanzania; Elizabeth Glaser Pediatric AID Foundation (EGPAF), P.O. Box 8822 Moshi, Tanzania

## Abstract

**Background:**

Psychosocial health problems, specifically depression during pregnancy, can have negative impact on birth outcomes, postnatal mental health of the mother, and infant health. Antenatal depression is more prevalent among women in low- and middle-income countries than among women in high-income countries. Risk factors for antenatal depression reported in the literature relate to pregnant women in South Asia. Consequently, this study assessed depression in pregnancy and related psychosocial risk factors among select pregnant women residing in Mwanza region, Northern Tanzania.

**Methods:**

We analysed data from 397 pregnant women recruited from three antenatal clinics for the period June—August 2013 for this cross-sectional study. Women provided data at one time point during their pregnancy by completing the Edinburgh Postnatal Depression Scale and a structured questionnaire assessing psychosocial, demographic, and behavioural risk factors related to antenatal depression. Multiple logistic regression analysis was performed to determine the relationship between risk factors examined and antenatal depression.

**Results:**

Overall, 33.8 % (*n =* 134) of pregnant women had antenatal depression. Pregnancy-related anxiety was associated with antenatal depression (odds ratio (OR) 1.36, 95 % confidence interval (CI) 1.23 to 1.5). Pregnant women with poor relationship with partner and low/moderate socio-economic status had the highest OR for antenatal depression (82.34, 95 % CI 4.47, 1516.60) after adjusting for other covariates. Pregnant women with poor relationship with partner and high socio-economic status had an OR of 13.48 (95 % CI 1.71, 106.31) for antenatal depression. “Reference” pregnant women were those with very good relationship with partner and high socio-economic status.

**Conclusions:**

High proportion of self-reported depression among select pregnant women attending antenatal clinics in Mwanza, Tanzania merit integrating depression assessment into existing antenatal care services. Health care providers need to assess pregnancy-related risk factors (pregnancy-related anxiety), socio-demographic factors (socio-economic status), and interpersonal risk factors (relationship with partner). Future research should appraise effectiveness of interventions that enhance partner relationships in reducing antenatal depression across all wealth distributions.

## Background

Antenatal depression, particularly mild depressive symptoms, may be overlooked by health care providers during antenatal assessment as it is likely attributed to emotional changes of pregnancy hormones [1]. Untreated antenatal depression can be associated with risk of postpartum depression, which has significant consequences for the health and well-being of the infant [2]. Antenatal depression is a public health concern due to its negative effect on the general health of the woman and its association with underutilization of antenatal care services, complications during pregnancy, negative experience of childbirth, adverse pregnancy outcomes (e.g., preterm birth, low birth weight, still birth, and birth asphyxia), and infant mortality and morbidity (i.e., poor infant growth and development) [3–7]. Moreover, the magnitude of the impact of antenatal depression varies across socioeconomic status [8, 9].

The World Health Organization (WHO) estimates that by the year 2020 depressive disorders will be the leading cause of global disease burden in women [10]. Moreover, the rate of depressive illness in women of reproductive age (15–49 years) is projected to be twice that of men in the same age range [10]. A systematic review of 21 studies on antenatal depression reported an overall prevalence of 10.7 %, though variability was noted in the individual studies (representing upper middle and high-income countries) [2]. Limited studies have been undertaken in low- and middle-income countries, exemplifying the disparity in health research expenditure, that is, the 10/90 gap (whereby less than 10 % of worldwide investments are directed to health research to address 90 % of global health problems) [11, 12]. The available literature, however, suggests higher rates of antenatal depression in low- and middle-income countries [11]. A systematic review of studies spanning 17 low- and middle-income countries reported a 15.6 % weighted mean prevalence of non-psychotic common perinatal mental disorders [12]. The weighted mean prevalence of maternal mental health disorders varied between low- and middle-income countries. In a systematic review of studies conducted in 8 African countries (Nigeria, South Africa, Uganda, Ethiopia, Morocco, Gambia, Zimbabwe, Malawi), Sawyer et al. reported a weighted mean prevalence of 11.3 % [13]. The study designs and measures of mental health differed between studies [13]. Furthermore, a study undertaken in a Tanzania peri-urban setting (an area transitioning between urban and rural; Chamazi and Mbagala), reported an antenatal depression rate as high as 39.5 % [14].

Sawyer et al.’s systematic review that examined mental health disorders of African women residing in Africa identified marital status—particularly single, separated/divorced and polygamous relations—and lack of support and conflicts with partner and within the family as risk factors for antenatal depression [13]. The contribution of socio-demographic and obstetric variables to mental health and well-being of African women residing in Africa were unclear [13]. Education, employment, and a healthy interpersonal relationship (i.e., kind and trusting) with a partner were reported to be protective factors [12]. Literature predominantly from high-income countries suggests that pregnancy-related anxiety, which is emotional states such as fears and worries related to the pregnancy itself [15], is a distinctive state from depression [16] but can coexist with depression [16–18]. Conceptual frameworks guiding exploration of risks and protective factors have not been consistent across studies, thus hindering pooling of data from various studies [12].

Freedom of choice is an integral characteristic for optimal development and self-actualization, as identified in person-centred theory [19]. The Tanzanian constitution, in principle, affords equals right to women by protecting them against gender-based discrimination [20]. Cultural norms and practices, however, are considered within the judiciary system, thereby discriminating against women and undermining their legal rights [20]. In Mwanza region (Ilemela, Magu, Misungwi), few women participate in decision making regarding their own health [21]. Lack of autonomy has been identified as predictor of antenatal depression among women in Pakistan, a low- and middle-income country [22]. Tanzania is a patriarchal society in which stereotypic notions of femininity and masculinity define gender roles. Poverty further exacerbates a father’s responsibility in providing financial resources despite his intentions to offer emotional and practical support in the care of an infant [23]. Social networks (e.g., family) were identified as an import resources to the well-being of the mother both during pregnancy and following birth [13, 23].

In Tanzania, antenatal care is focused more on physical health in pregnancy, paying little attention to psychosocial health of pregnant women [24]. Thus, women with antenatal depression may not be identified during prenatal period to initiate psychosocial interventions to prevent or mitigate the adverse consequences associated with antenatal depression. The social and environmental context is important to consider, as they have been predominantly associated with antenatal depression [25]. Understanding the enormity and nature (i.e., risk factors) of antenatal depression is imperative to improve maternal mental health in order to achieve Millennium Development Goals 5 and 4 of promoting health and well-being of mother and child, respectively. We aimed to determine the proportion of antenatal depression among select pregnant women in Mwanza region, northern Tanzania with antenatal depression and explore risk factors associated with antenatal depression.

## Methods

### Study design, setting and participants

We conducted a cross-sectional study from June to August 2013 in Mwanza city, which has a total of 17 government health facilities (3 hospitals, 2 health centres, and 12 dispensaries) that provide comprehensive antenatal care services. Annually, 20,427 pregnant women receive antenatal care from these facilities during their first (42 %), second (73 %), and third (29 %) trimesters [26]. Nyamagana (hospital), Makongoro (health centre), and Buhongwa (dispensary) were purposefully selected to include at least one facility at each level of health care to increase likelihood of a diverse sample. Nyamagana is the only public hospital in the area, and given the time constraints for data collection, Makongoro and Buhongwa were selected based on their high antenatal care attendance rates [26]. Every day, approximately 40–50 pregnant women seek antenatal care services from Nyamagana and Makongoro, while 20–30 pregnant women seek antenatal care services from Buhongwa [26].

A sample size of 369 was estimated using Kish Leslie’s formula [27], with a 40 % proportion of antenatal depression based on a local study (Dar es Salaam, Tanzania) [14] and a margin of error set at 0.05 and a 95 % confidence interval (CI). To account for potential refusal and incomplete data, 400 pregnant women were approached at any time during their pregnancy using a cross-sectional design with systematic sampling technique (e.g., every third pregnant women) and all agreed to participate in the study (Nyamagana, *n =* 170; Makongoro, *n =* 170; Buhongwa, *n =* 60).

Pregnant women who attended selected antenatal clinics and were registered at these clinics during the study period were eligible. In 2006, sentinel surveillance at antenatal clinics in Magu district in Mwanza revealed a greater than 7 % prevalence of human immunodeficiency virus with no variation across community-based cohorts in the region [28]. As disclosure rates vary and significant stigma is attached with the diagnosis of human immunodeficiency virus, no attempts were made to elicit pregnant women’s infection status. No pregnant women were excluded because they presented with urgent health care needs such as febrile conditions and obstetric emergencies such as vaginal bleeding requiring referral to health services. This was not surprising, as the antenatal clinics do not provide medical treatments.

A brief introduction regarding the study team, study scope, and benefits and potential risks of participating was given to staff, specifically the nurse in charge, at the antenatal clinic sites. The nurse in charge approached eligible participants during the health education session given at the facility. Those indicating a willingness to participate were approached by a researcher (MR) or research assistants who were qualified health care providers (e.g., nurse midwife, registered nurse/psychiatrist, public health nurse). The registered nurse/psychiatrist and public health nurse were trained counsellors. The research assistants underwent two days of training about the study purpose, design, tools, and protocol to manage mental health crisis in participants.

### Data collection

The Edinburgh Postnatal Depression Scale (EPDS) was used to assess participant’s depressive symptoms during pregnancy. The EPDS is the most reliable and widely used measure of depression during pregnancy in high-income countries and in African countries [13, 29, 30]. The 10-item tool, which generates a score from 0 to 30, has fewer somatic cognitive items, thus will not overestimate the proportion of depression [2]. In a high-risk population of pregnant women (i.e., human immunodeficiency virus) the sensitivity and specificity of the EPDS was reported at 69 % and 78 %, respectively. Women were correctly classified using a cut-off of  ≥ 13 [29]. Women with a cut-off of  ≥ 13 on the EPDS were categorized as having antenatal depression.

The socio-ecological framework for health promotion programs [31], specifically the intrapersonal (e.g., age, education, marital status, socio-economic status, pregnancy-related anxiety, gravidity, history of loss of child, nature of previous delivery) and interpersonal levels (e.g., relationship with partner, social support from family members, involvement in decision making), was used to guide assessment of social and environmental factors associated with antenatal depression. Nature of previous delivery was classified as normal (i.e., spontaneous vaginal delivery with a live born infant), abnormal (i.e., caesarean section, forceps, vacuum, postpartum haemorrhage, stillbirth, etc.), and not applicable (i.e., first pregnancy). Pregnancy-related anxiety was assessed using the pregnancy-related anxiety 10-item tool (4-point Likert scale) evaluating a woman’s feeling regarding her health during pregnancy, the health of her fetus/infant, and labour and delivery. Item ratings were summed using reverse scoring where appropriate to generate a cumulative score ranging from 10 to 40 [32]. Given social norms, it was not feasible to determine individual or household income per month or per annum. Consequently, socio-economic status was assessed through inquiry regarding living standards (e.g., type of roof of house, access to water and electricity, number of meals per day), selected assets (e.g., car, motorcycle, bicycle), and other wealth status (e.g., employment) [33]. Standardized weight or factor scores assigned to each response [33] were totalled to categorize wealth tertile of the individual participants as low (score 0–5) to moderate (score 6–11) and high (12–18) given the distribution of data.

Three factors related to the interpersonal level of the socio-ecological framework were examined, namely, involvement in decision-making, relationship with partner, and social support from family. To limit participant burden women were asked to respond to three closed-ended questions: (a) Are you involved in decision-making regarding your pregnancy? (always, sometimes, never); (b) How would you describe your relationship with your partner? (very good, fair, poor); and (c) Do you feel supported by members of your family? (very much supported, fairly supported, not supported).

The questionnaire was piloted with 10 pregnant mothers from the Mkolani antenatal clinic, which was not part of the study setting, before being implemented for this study. Piloting the questionnaire helped to ensure that the Swahili translation of the questionnaire was valid (i.e., questions were easily understood by the participants). Double-barrelled questions (e.g., reference to partner and social support) were modified. The researcher (Sr M) or research assistants carried out face-to-face individual interviews to assist women in completing the questionnaire, which included assessment risk factors at both individual and interpersonal levels. All field data was reviewed for completeness; women provided missing information (e.g., gestational age, parity) before they left the clinic.

### Data analysis

Data were analysed using R [19]. Numerical data were summarized using mean (standard deviation) or medians (interquartile-range), while categorical data were summarized using frequencies and proportions. Data were collapsed from interval to nominal level (e.g., age, socio-economic wealth tertile to examine differential age distribution of antenatal depression or used as a proxy measure for income index such as low, moderate, and high) or categories collapsed (e.g., marital status as the observed value was less than 5 in some cells) to permit data analysis. The primary aim of this study was to determine the proportion of antenatal depression among select pregnant women, which was calculated by dividing the number of women categorized as having antenatal depression (score of  ≥ 13 on EPDS) by the total number of women participating in the study multiplied by 100.

To explore the relationship between antenatal depression and other covariates or explanatory variables, multiple logistic regression analysis was undertaken. Given that the interaction of some variables presented cells with zero counts, Firth’s penalized-likelihood logistic regression was used [34]. Based on the statistical rule of thumb [35], the number of successes and failures in the outcomes in our data (see Results section) allowed us to estimate a maximum of 13 coefficients. Based on a combination of strength of evidence in the literature and scientific judgement, 4 covariates—marital status, social support from family, relationship with partner and pregnancy-related anxiety—were deemed as main exposures and kept throughout the modelling process regardless of their statistical significance. We used recursive partitioning with classification trees [36] as an exploratory tool to assess which other variables from the data seemed to play a role in the relationship to antenatal depression; these were included in the model and assessed for confounding and statistical significance. We assessed for confounding effect from covariates by determining if there was a change of 10 % or more in the regression coefficients of the main exposures. Interactions, which were gleaned from the literature to be important (e.g., relationship with partner with socio-economic status, pregnancy-related anxiety with socio-economic status), were included in the model to test for their statistical significance. A significance level alpha of 0.05 was used. The final regression model was constructed by including the main exposures, the inclusion of the interaction terms deemed to be statistically significant, and the inclusion of extra covariates identified in the classification tree that were confounders and/or statistically significant.

### Ethical considerations

Ethics approval was obtained from the Joint Bugando Medical Centre and Catholic University of Health and Allied Sciences ethics clearance committee. Permission to conduct the study was obtained from the Regional and District authorities in Mwanza, Region. Informed consent was obtained after providing participants information about the study, including purpose, expectations regarding participation, foreseeable risks and benefits, and the voluntary nature of the participation without impact on care received at the facility. At the request of some participants, the researcher rather than the research assistants interviewed the participants so as to protect their privacy. Women were interviewed in a private room and no family members were allowed to accompany the participants to permit freedom of expression, privacy, and confidentiality. Four participants were not able to read the consent form, thus the content was read to them and they provided a thumbprint instead of a signature to indicate consent.

## Results

### Study sample

Of the 400 women who were approached and enrolled in the study 397 pregnant women were included in the analyses, as 3 pregnant women had incomplete data. Their median age was 26 years (range 18 to 42 years). The majority of the pregnant women (*n =* 316, 79.6 %) were married and only a few (*n =* 15, 3.8 %) were single mothers. Their male partners were older, with median age of 31 years (range 19 to 60 years). Many of the pregnant women (*n =* 195, 49.1 %) were in their second trimester, while fewer (*n =* 51, 12.9 %) were in their first trimester. A total of 137 (34.5 %) of the women were primigravida. Of the multiparous women (*n =* 260, 65.5 %), a greater number (*n =* 225, 86.5 %) described their previous pregnancy as “normal.” History of loss of a child was reported by 61 (15.4 %) of pregnant women (or 23.5 % of multiparous women). The median number of children was 1, while the range was 8. More than half of the pregnant women had primary level education (*n =* 216, 54.4 %), and only 24 (6.1 %) had no formal education. Over half (*n =* 217, 54.7 %) of the pregnant women were classified as being in the low to moderate wealth tertiles (0 to 11 score; see Table 1).

**Table 1:** Characteristics of pregnant women enrolled from antenatal clinics in Mwanza Region, Tanzania

		Antenatal depression	
Participant’s characteristics	Total (*N =* 397) n (%^a^)	Yes (*n =* 134) n (%)	No (*n =* 263) n (%)
Age of pregnant women (years)			
18–25	186 (46.9)	66 (49.3)	120 (45.6)
26–35	176 (44.3)	57 (42.5)	119 (45.3)
>35	35 (8.8)	11 (8.2)	24 (9.1)
Trimester			
1st trimester	51 (12.9)	21 (15.7)	30 (11.4)
2nd trimester	195 (49.1)	67 (50.0)	128 (48.7)
3rd trimester	151 (38.0)	46 (34.3)	105 (39.9)
Gravidity			
Primigravida	137 (34.5)	43 (32.1)	94 (35.7)
Multigravida (2–4 times)	205 (51.6)	71 (53.0)	134 (51.0)
Grand multigravida (≥5 times)	55 (13.9)	20 (14.9)	35 (13.3)
History of child loss			
Yes	61 (15.4)	26 (19.4)	35 (13.3)
No	199 (50.1)	65 (48.5)	134 (51.0)
Not applicable – first pregnancy	137 (34.5)	43 (32.1)	94 (35.7)
Nature of previous pregnancies			
Normal	225 (56.7)	70 (52.2)	155 (58.9)
Abnormal	35 (8.8)	21 (15.7)	14 (5.3)
Not applicable	137 (34.5)	43 (32.1)	94 (35.7)
Age of partner (years)			
≤30	198 (49.9)	69 (51.5)	129 (49.1)
31–40	141 (35.5)	49 (36.6)	92 (35.0)
>40	58 (14.6)	16 (11.9)	42 (16.0)
Marital status			
Married	316 (79.6)	94 (70.2)	222 (84.4)
Cohabiting	66 (16.6)	28 (20.9)	38 (14.5)
Single	15 (3.8)	12 (9.0)	3 (1.1)
Level of education			
No formal education	24 (6.1)	8 (6.0)	16 (6.1)
Primary	216 (54.4)	78 (58.2)	138 (52.5)
Secondary	112 (28.2)	37 (27.6)	75 (28.5)
Higher education	45 (11.3)	11 (8.2)	34 (12.9)
Social economic status			
High wealth tertile	180 (45.3)	49 (36.6)	131 (49.8)
Low to moderate wealth tertiles	217 (54.7)	85 (63.4)	132 (50.2)
Family support			
Very much supported	225 (56.7)	35 (26.1)	190 (72.2)
Fairly supported	75 (18.9)	36 (26.9)	39 (14.8)
Not supported at all	97 (24.4)	63 (47.0)	34 (12.9)
Pregnancy decision			
Always involved	211 (53.1)	24 (17.9)	187 (71.1)
Involved sometimes	139 (35.0)	69 (51.5)	70 (26.6)
Never involved	47 (11.8)	41 (30.6)	6 (2.3)
Partner relations			
Very good	219 (55.2)	22 (16.4)	197 (74.9)
Fairly good	128 (32.2)	63 (47.0)	65 (24.7)
Poor	50 (12.6)	49 (36.6)	1 (0.4)
Pregnancy-related anxiety			
(median [IQR])	14 [6]	18 [4]	13 [4.5]

*IQR* Interquartile Range

^a^Percentages might not add up to 100 due to rounding

### Proportion of our sample with antenatal depression

Of the 397 pregnant women included in the analyses, 134 (33.8 %) were categorized as having antenatal depression (they scored 13 points or higher on the EPDS) and were referred for counselling. Of the 134 women with antenatal depression, 26 accepted brief counselling at the time of the visit. Two participants who reported self-harm ideation were referred to a nurse psychiatrist at Makongoro Health Centre for assessment and management. All counselling services were offered free of charge. Information about mental health during pregnancy and resources available to support women were shared with all participants after the face-to-face interview.

### Relationship between risk factors and antenatal depression

Intrapersonal risk factors appraised included age, education, marital status, socio-economic status as determined by socio-economic wealth tertiles, pregnancy-related anxiety, gravidity, history of loss of child, and nature of previous delivery (see Table 1). Interpersonal-level risk factors appraised included involvement in decision-making, relationship with partner and social support from family (see Table 1). The classification tree yielded four covariates, namely relationship with partner, pregnancy-related anxiety, involvement in decision-making, and social support from family. The final regression model included marital status, pregnancy-related anxiety, social support from family, involvement in decision-making, and the relationship with partner and socio-economic status interaction. The regression coefficients with their p-values and odds ratios (OR) can be seen in Table 2.

**Table 2:** Final regression model: regression coefficients, p-value, and odds ratio

Participants’ characteristic	Regression coefficient	p-value	OR [95 % CI]
Marital status			
Married	Reference	Reference	Reference
Cohabiting	0.27	0.52	1.31 [0.57, 3.02]
Single	−0.58	0.59	0.56 [0.07, 4.72]
Family support			
Very much supported	Reference	Reference	Reference
Fairly supported	0.43	0.3	1.54 [0.68, 3.47]
Not supported at all	0.34	0.43	1.41 [0.60, 3.28]
Pregnancy decision			
Always involved	Reference	Reference	Reference
Involved sometimes	0.6	0.2	1.82 [0.73, 4.52]
Never involved	1.38	0.06	3.97 [0.96, 16.45]
Pregnancy-related anxiety	0.31	<0.001	1.36 [1.23, 1.50]
Relationship with partner			
Very good	Reference	Reference	NA
Fairly good	0.51	0.37	NA
Poor	2.6	0.01	NA
SES			
High wealth tertile	Reference	Reference	NA
Low to moderate wealth tertiles	−0.55	0.25	NA
Relationship with partner x SES interaction			
Very good relationship x high SES	NA	NA	Reference
Very good relationship x low to moderate SES	NA	NA	0.58 [0.23,1.47]
Fairly good relationship x high SES	NA	NA	1.66 [0.55, 5.02]
Fairly good relationship x low to moderate SES	1.37	0.03	3.77 [1.30, 10.98]
Poor relationship x high SES	NA	NA	13.48 [1.71, 106.31]^a^
Poor relationship x low to moderate SES	2.36	0.17	82.34 [4.47, 1516.60]^a^

*OR* odds ratio, *CI* confidence interval, *NA* not applicable, *SES* socio-economic status

^a^These CI are too wide given low frequencies by outcome in these categories

The adjusted OR of antenatal depression among pregnant women was 1.37 (95 % CI 1.23, 1.5) for one unit increase in pregnancy-related anxiety. For the interaction term, “reference” pregnant women were those with a very good relationship with partner and high socio-economic status. The adjusted OR of antenatal depressive among pregnant women with a poor relationship with partner and high socio-economic status was 13.48 (95 % CI 1.71, 106.31), while the adjusted OR of antenatal depressive among pregnant women with a fairly good relationship with partner and low/moderate socio-economic status was 3.77 (95 % CI 1.3, 10.98). Pregnant women with a poor relationship with partner and low/moderate socio-economic status had the highest OR for antenatal depression (82.34, 95 % CI 4.47, 1516.60) after adjusting for all other covariates. Although all the other covariates were included in the final model, given their clinical importance, none of them had a statistical significant effect on the odds of depression.

## Discussion

Overall, 134 (33.8 %) of pregnant women in the study had a score of 13 or greater on the EPDS, thus identified as having antenatal depression. The Sawyer et al. systematic review of studies conducted in 8 African countries reported a weighted mean prevalence of antenatal depression as 11.3 % (range 4.3 % to 17.4 %) [13]. Disparity in proportion of women who experience antenatal depression across countries, despite being within Africa, may be explained by differences in study design, tools used to measure antenatal depression, cut-offs established to categorize pregnant women as depressed or not depressed, and differences in social and environmental risk factors contributing to antenatal depression. The proportion of antenatal depression in our sample is more consistent with a study undertaken in a peri-urban setting (Chamazi and Mbagala) in Tanzania that reported an antenatal depression proportion of 39.5 % [14]. Our study combined urban and peri-urban health centres to increase diversity of the sample; distinctive socio-environmental risk factors between the 2 settings–urban and peri-urban–may explain the variability in proportion of antenatal depression. Our study used the EPDS while the Tanzanian study used the Hopkins Symptom Checklist; although demonstrated to be reliable and valid in a Tanzanian population, the later was found to be less sensitive in discriminating levels of depression [37]. Furthermore, the EPDS focuses on cognitive items rather than somatic symptoms (e.g., fatigue, difficulty sleeping) that may overestimate the proportion or severity of depression [2], particularly in endemic malaria settings like Tanzania.

Both animal and human studies demonstrate that psychological responses to socio-environmental stressors vary across pregnancy, with the magnitude of response being more pronounced early in pregnancy [38–40]. Consequently, timing of assessment of antenatal depression (i.e., trimester of pregnancy at the time of enrolment) was included in the classification tree; however, findings suggested that it did not influence the outcome (i.e., antenatal depression). Animal and human studies demonstrate that psychological and biological responses to psychosocial distress vary across pregnancy, with more blunted responses as pregnancy progresses [38–40]. Chronic stress experienced by pregnant women in low- and middle-income countries may, however, alter psychological and biological responses [11] in that there is no blunting of response over the course of pregnancy.

Pregnancy-related anxiety, emotional states such as fears and worries related to the pregnancy itself [15], was an independent risk factor for antenatal depression. In Tanzania, pregnancy-related mortality (maternal and fetal/newborn) is high as a result of three delays: decision-making regarding seeking care, reaching or accessing appropriate health care facility, and inadequate care (i.e., poor quality) or available resources [41, 42]. Anxiety during pregnancy has been attributable to previous difficult delivery, specifically caesarean section or instrumental deliveries, and previously complications during pregnancy [43]. Consistent with a study undertaken in another low- and middle-income country (Anuradhapura, Sri Lanka), age and education were not associated with antennal depression [44].

Marital status, specifically single, separated/divorced, and polygamous relations, have been identified as a risk factor for antenatal depression in a systematic review examining mental health disorders of African women residing in Africa [13]. Given the distribution of data, categories related to marital status were collapsed to married, cohabiting, and single/separated/widow status; no women reported being divorced. Marital status was not an independent risk factor for antenatal depression in our study. Socio-economic status (as measured by wealth tertiles) modified the relationship between depression and the relationship with partner. A qualitative study exploring postpartum experiences of first time fathers in Ilala suburb of Dar-es-Salaam, Tanzania described fathers as sensitive to the well-being of the mother and infant [23]. The competing demands of securing financial resources for the family hindered their engagement in caring responsibilities [23].

The Tanzania Demographic and Health Survey 2010 [45] reported that 82.3 % of women aged 15 to 49 years (i.e., childbearing age) are employed, while 79.4 % of men aged 15 to 49 years are employed. Cost of care associated with pregnancy, delivery, and a new infant may strain financial resources even further. Since more women than men work, earning potential of the family can be impacted during childbearing years. Limited studies have examined the contribution of socio-economic status to mental health and well-being of African women residing in Africa [13]. Adewuya [25] found no association between socio-economic status and antenatal depression. The literature is controversial in that studies examining antenatal depression and pregnancy outcomes or adverse obstetric outcomes suggest that the magnitude of the impact of antenatal depression varies across socioeconomic status [8, 9].

In our study, it was challenging to find research assistants who were comfortable asking sensitive questions and had the knowledge and expertise in counselling pregnant women with mental health problems. We therefore relied on health care providers at the antenatal care settings, who had a background in psychiatric nursing and some experience with counselling, to assist with data collection. We negotiated with the nurse in charge to release these health care providers for a 2-day training and permit flexibility in scheduling so they could assist with data collection. Some pregnant women were apprehensive while others preferred to be interviewed by a health care provider involved in their antenatal care. Consequently, flexibility was maintained with respect to who interviewed the pregnant women so that they would be inclined to respond openly and honestly and remain in the study. When planning future studies, consideration will need to be given to the individual preferences of pregnant women with regards to who interviews them for data collection related to sensitive aspects of their life (e.g., mental health), as well as the competency of those involved in data collection.

Our study was health-facility based (hospital, health centre, and dispensary), thus pregnant women with depression who do not seek antenatal care services would not be captured. Gender-based risk factors such as experiencing intimate partner violence were not examined in our study as social norms do not permit discussion of domestic matters in public. Other limitations include the cross-sectional study design, which precludes interpretation regarding the direction of relationships (e.g., pregnancy-related anxiety contributing to antenatal depression versus antenatal depression contributing to pregnancy-related anxiety). Recall bias and social desirability are threats to the internal validity of this study. EPDS is a screening tool and not a diagnostic tool and we have not validated the Swahili version of the EPDS in the local context of Mwanza, Tanzania. Lastly, we did not use a standardized instrument to measure social support, which hinders the inferences we can make. Despite these limitations, we add new knowledge to the field as few studies have examined antenatal depression and risk factors of antenatal depression in low- and middle-income countries. Given social, cultural, and environmental differences, it would be prudent not to generalize the findings of our study. Future studies need to examine the impact of psychosocial interventions in reducing poor pregnancy outcomes and improving maternal and infant health in the postpartum period.

## Conclusions

Our findings suggest that high proportion of antenatal depression is prominent among select pregnant women seeking care from Nyamagana District Hospital, Makongoro Health Centre, and Buhongwa Dispensary. Risk factors included pregnancy-related anxiety, socio-economic status, and relation with partner. Comprehensive antenatal care services including timely identification and management of pregnant women with antenatal depressive is of paramount importance to achieving better pregnancy and infant outcomes (i.e., mortality and morbidity). A collaborative care approach between antenatal care services and mental health care practitioner will ensure that women who report self-harm are managed appropriately.

## Abbreviations

CI: Confidence interval
EPDS: Edinburgh Postnatal Depression Scale
IQR: Interquartile range
NA: Not applicable
OR: Odds ratio
SES: Socio-economic status
WHO: World Health Organization
